# Effects of plant-based fermented broth on rumen fermentation, gastrointestinal development, and microbial populations in fattening lambs

**DOI:** 10.3389/fvets.2025.1584930

**Published:** 2025-09-12

**Authors:** Lu Zhang, Huiwen Zhang, Lianjie Song, Yongliang Li, Jianjun Guo, Feng Li, Bosen Li, Wei Chen, Yuqing Liu, Haitong Yang, Jianjie Li, Yuhong Gao, Xinsheng Sun

**Affiliations:** ^1^College of Animal Science and Technology, Agricultural University of Hebei, Baoding, Hebei Province, China; ^2^Veterinary Drug Administration, Chengde, Hebei Province, China; ^3^Chengde Academy of Agricultural and Forestry Sciences, Chengde, Hebei Province, China; ^4^College of Information and Technology, Agricultural University of Hebei, Baoding, Hebei Province, China

**Keywords:** fermentation, fattening lamb, rumen fermentation, digestion, microstructure, microbiota

## Abstract

**Introduction:**

Digestive dysfunction in lamb fattening has been a concern in recent years. The objective of this study was to investigate the effects of a fermentation broth (FB) derived from eight plants on rumen fermentation, gastrointestinal development, and microbial community composition in fattening lambs through *in vitro* and *in vivo* experiments.

**Methods:**

*In vitro* rumen fermentation was performed with six FB: diet ratios (mL/g): 0, 1:125, 1:250, 1:500, 1:1000, and 1:2000. Fermentation parameters and nutrient disappearance rates were measured over 48 h. Based on the results of the *in vitro* test, two optimal doses of FB (1:500 and 1:1000) were selected for further *in vivo* testing over a 120-day period. A total of 90 weaned small-tailed Han lambs were assigned to three groups, receiving FB in drinking water at 0 (control), 1:500, or 1:1000 (vol/vol).

**Results:**

The results showed that three parameters of gas production (GP), including fast-degradable fraction, slow-degradable fraction, and theoretical GP, were significantly higher (*p* < 0.05) in the 1:500 and 1:1000 groups compared with other groups. *In vivo* tests further showed that FB supplementation at 1:500 and 1:1000 improved rumen microstructure (papilla length, papilla surface, mucosa thickness, and muscle thickness) and jejunal microstructure (villus height, villus surface, and muscle thickness). Moreover, two rumen bacterial indices, PD_whole_tree (*p* = 0.06) and Shannon (*p* = 0.07), demonstrated increasing trends in both FB groups relative to the control group. In the rumen, 1:500 FB supplementation increased the abundance of Firmicutes (*p* < 0.01) and decreased Bacteroidota abundance (*p* < 0.01). In the jejunum, 1:500 FB supplementation decreased the abundance of Euryarchaeota and *Methanobrevibacter* (both *p* = 0.02) while increasing the abundance of Actinomycetes and *Aeriscardovia* (both *p* = 0.01) compared to the control.

**Discussion:**

In summary, FB supplementation in lambs’ drinking water at a ratio of 1:500 (FB: diet, mL/g) improved rumen fermentation and promoted microecological balance.

## 1 Introduction

Human health-related antibiotics have been banned as feed additives in the European Union and China due to concerns over drug residues and antibacterial resistance (1). This has made the search for effective antibiotic alternatives a persistent challenge in sustainable animal husbandry. Natural products (e.g., Chinese herbal medicines and plant extracts) have been widely used in monogastric animals such as chickens (2), pigs (3), and rabbits (4) due to their safety and low residue levels. Their use as feed additives in ruminants such as cows (5) and sheep (6) is also increasing; however, they still present certain limitations as antibiotic alternatives. Recent studies suggest that modified fermentation processes can enhance the biological activity of fermented materials by producing more probiotics and bioactive components (e.g., polysaccharides and flavonoids), thereby improving gastrointestinal digestion and nutrient absorption. Previous studies have demonstrated that plant fermentation products, such as fermented alfalfa (7) and fermented astragalus (8), promote animal growth performance when incorporated into feed. Nevertheless, little information is available regarding the effects of plant fermentation on gastrointestinal microbial communities (9).

Recently, lamb fattening has been developing rapidly to meet the growing demand for mutton in China, with intensive indoor-fattening systems widely adopted. However, Hoque et al. (10) reported that such feeding patterns may disrupt the gastrointestinal environment, reducing digestibility and compromising lamb health. Although significant progress has been made—for example, dietary probiotics and traditional Chinese medicines can improve lamb growth to some degree (11, 12)—maintaining gastrointestinal balance and enhancing the digestion of nutrients remain major challenges in the fattening industry of lambs. In this study, we evaluated fermented broth (FB) prepared from a mixture of forage grasses and Chinese herbal medicines for its effects on gastrointestinal development and microbiota in fattening lambs. FB was supplemented through drinking water, a delivery method that ensures uniform distribution and stable bioactivity (13, 14).

## 2 Materials and methods

### 2.1 Preparation of fermentation broth

A total of eight plants were used in the present study, including four kinds of forage grasses (alfalfa, ryegrass, sudangrass, and hybrid giant napier) and four kinds of traditional Chinese medicines (*Astragali Radix*, *Poria*, *Platycladus orientalis*, and *Folium Isatidis*). First, the plants were cleaned, minced, and dried at room temperature. After being soaked for 1 h with distilled water, each plant material was boiled for another 30 min according to 1 g of material (dry matter (DM) basis) into 1 mL of distilled water and then filtered to collect the filtrate. All filtrates of eight plants were mixed thoroughly in equal proportion to obtain the mixture of all materials (M0) and then subpackaged into eight aliquots. Each individual filtrate was added to M0 at a volume ratio of 1:10 (vol/vol), followed by the incorporation of 12% (g/mL) brown sugar and 1% *Lactobacillus* (1.7 × 10^5^ cfu/mL). After thorough mixing, the mixture was transferred to a closed vessel, and its pH was adjusted to 4.0 using 2 mol/L NaOH or HCl as needed. Fermentation was then conducted at 26–27 °C for 3 months under controlled conditions to obtain the first batch of individual FB. Eight individual FBs were mixed in equal proportions to produce the first mixture (M1). Subsequently, each individual FB was added to M1 at a 1:10 (vol/vol) ratio, and the same fermentation process was repeated to generate the second batch of individual FB, which were then combined to form the second mixture (M2). Finally, M2 was mixed with M0 in equal proportions and fermented for 3 months under the same conditions to produce the final FB needed in this experiment.

### 2.2 *In vitro* rumen fermentation, experimental design, sample collection, and measurement

Preparation of artificial rumen fluid. Rumen fluid was collected from healthy fistula lambs raised in the College of Animal Science and Technology at Hebei Agricultural University in China. Approximately 2 L of rumen fluid was taken from the fistula sheep, squeezed, filtered by four layers of gauze, and transferred into an anaerobic vacuum bottle preheated to 39 °C. Then, carbon dioxide (CO_2_) was added continually into the vacuum bottle for 5 min. The collected rumen fluid was mixed with anaerobic buffer prepared at the ratio of 1:3 to obtain artificial rumen fluid according to the method published by Menke et al. (15). Before the mixture, the buffer was placed at 39 °C in a water bath oscillator (THZ-82A, Shanghai Jinpeng Analysis Instrument Co., Ltd., China) and added CO_2_ continually for 1 h. Meanwhile, the pH value of the buffer was adjusted to 6.8 using a pH meter by adding 2 mol/L NaOH. Then, a reducing agent, including Na_2_S·9H_2_O and NaOH, was added to the buffer to remove all residual oxygen. When the color of the buffer was turned light yellow, the butter was under anaerobic conditions.

*In vitro* rumen fermentation design. This experiment was performed in a completely randomized design. Based on our pre-experiment results, the test included six groups with five replicates for each group (a fermentation vessel of 250 mL per replicate). The five treated groups included 1:2000 (FT1), 1:1000 (FT2), 1:500 (FT3), 1:250 (FT4), and 1:125 (FT5) by volume/mass (mL/g, DM basis), and the control group (C) was not supplemented with FB. The basal diet was prepared based on the concentrate: roughage of 65:35 for fattening lamb. The detailed fermentation process was as follows: 1.0 g of diet was put into each fiber bag and sealed, then packed into the fermentation vessel and sealed to keep a vacuum condition. A total of 100 mL of the above artificial rumen fluid and 1 mL of diluted FB, according to the assigned FB dose for each treatment group (with 1 mL of distilled water used for the control group), were injected into individual fermentation vessels using an injector. Fermentation was then conducted at 39 °C for 48 h in a water bath oscillator at 45 rpm. The composition of the basal diet and its nutrient composition (DM basis) are provided in Table 1.

**Table 1:** Composition of basal diet and nutrient composition (dry matter basis).

Ingredient	% of DM	Nutrient composition^2^	% of DM
Whole-plant corn silage	35.41	ME/(MJ/kg)	11.51
Corn	37.78	CP	14.24
Soybean meal	9.00	NDF	24.33
Cotton meal	4.50	ADF	13.25
DDGS	4.50	Ca	0.56
Wheat middling	1.49	P	0.33
Spraying corn husk	2.72		
Premix^1^	2.00		
NaCl	0.60		
Ca(HCO_3_)_2_	1.20		
NaHCO_3_	0.80		
Total	100.00		

1 The premix provided the following per kg of diet: VD3 (Vitamin D3) 1,500 IU, VA (Vitamin A) 3,200 IU, VE (Vitamin E) 680 IU, Fe 30 mg, I 0.8 mg, Zn 30 mg, Se 0.3 mg, Cu 10 mg, Co 0.25 mg, and Mn 25 mg.

2 ME was a calculated value, while the others were measured values.

Fermentation parameter measurement. At 2, 4, 6, 8, 10, 12, 24, 36, and 48 h of fermentation, all gasses in each vessel were extracted using an injector and recorded. According to the model of gas production (GP) reported by Orskov and McDonald (16), the GP for each time period was calculated as follows:

${\mathrm{GP}}_{t}=a+b\;(1-e^{-\mathrm{ct}})$ ,

where GP*_t_* is GP at time *t* (mL); t is fermentation time (h); *a* is GP of fast-degradable fraction (mL); *b* is GP of slow-degradable fraction (mL); *c* is the rate of GP of slow-degradable fraction (%/h).

At the end of the fermentation process, the pH value of artificial rumen fluid in each vessel was detected. The 10 mL of rumen fluid in each vessel was taken and frozen at −80 °C until further analysis for volatile fatty acid (VFA), ammonia nitrogen (NH_3_-N), and microbial protein (MCP). VFA concentrations (acetic acid, propionic acid, butyric acid, etc.) were determined by gas chromatography (17). NH_3_-N and MCP concentrations were measured by the methods of indophenol colorimetry (18) and coomassie brilliant blue staining (19), respectively.

Nutrient disappearance rate measurement. At the end of fermentation, the fiber bags filled with fermentation residues were washed with distilled water and dried in an oven at 65 °C for approximately 4 h until constant weight. The disappearance rates of DM, crude protein (CP), neutral detergent fiber (NDF), and acid detergent fiber (ADF) were determined according to the methods from Bodas et al. (20). Disappearance rate (%) = 1 - nutrient content in fermentation residue/nutrient content in diet. The contents of DM and CP in diets and fermentation residue were detected according to the method of Lee (21). The NDF and ADF were determined by the method from Soest (22).

### 2.3 Animals and treatments

According to the results of the above *in vitro* rumen fermentation, two optimal doses of FB supplements (1:500 and 1:1000, mL/g, DM basis) were selected for further experimentation in animal production. In this experiment, the method of adding FB to drinking water was conducted. Based on the feed-to-water ratio of 1:1 (volume/mass, mL/g, DM basis) reported in our previous study by Zhao et al. (23), the ratio of FB to drinking water was equivalent to the ratio of FB to the diet. A total of 90 weaned small-tailed Han lambs (27.12 ± 0.55 kg initial body weight, 3-month age) were used and allocated into three groups in a completely randomized design. The lambs in two treated groups were offered FB at 1:1000 for the low-dose group (T1) and 1:500 for the high-dose group (T2) based on the ratio of FB to drinking water (volume basis), and those in the control group (C) were not provided FB in drinking water. There were 30 lambs in each group, with five replicates per group (six lambs per replicate/pen). The entire test lasted 130 d, including a 10-d adaptation period and a 120-d experimental period. All lambs were provided with drinking water twice a day at approximately 0800 and 1,600 h, and it was guaranteed that there was residual water in the troughs for the lambs to drink freely for 24 h. The quality of drinking water must meet the requirements in China for drinking water for livestock and poultry (China Standard, NY 5027) (24). A total mixed ration (TMR) was available ad libitum and offered twice a day at approximately 0700 and 1,500 h. The composition and nutritional level of the TMR are presented in Table 1. On the last day of the experiment, prior to early morning feeding, six lambs from each group were randomly selected for slaughter by the method of bloodletting through the jugular vein. Samples were then collected for subsequent analysis.

### 2.4 Collection, measurement, and analysis of rumen liquid and jejunum content samples

A sample of rumen fluid (50 mL) from each slaughtered lamb was collected and filtered through four layers of gauze. Part of the filtrate (20 mL) was used to measure the pH value on the spot using a pH meter (SevenDirect SD20 Kit, Mettler Toledo, China). The 2 mL of filtrate from each slaughtered lamb (four lambs per group) was stored in liquid nitrogen for further measurement of the microbial community. Another 10 mL of filtrate from each slaughtered lamb was added to 0.1 mL HCl (6 mol/L) and stored at −20 °C until analysis of NH_3_-N by the method of indophenol colorimetry (18). The rest of the filtrate was stored at −80 °C until analysis of VFA by gas chromatography (17). Additionally, samples of jejunum content (2 mL) from each slaughtered lamb (four lambs per group) were also taken from the middle part of the jejunum and preserved in liquid nitrogen for the measurement of microbial community. The samples for microbial community analysis were sent immediately to Beijing Allwegene Gene Technology Co., Ltd. for further measurement after slaughter. Simply, DNA was extracted from rumen fluid and jejunal content samples. Following assessment of DNA integrity and purity via 1% agarose gel electrophoresis and spectrophotometry. All 16S rRNA genes in the hypervariable region of V3 to V4 from bacteria were amplified by the primers of 341F (5′-CCTACGGGRBGCASCAG-3′) and 806R (5′-GGACTACHVGGGTWTCTAAT-3′) and sequenced using the Illumina Miseq PE300 platform by high-throughput sequencing technique. The sequence was uploaded to the SRA database in the National Center for Biotechnology Information (NCBI, www.ncbi.nlm.nih.gov, PRJNA1047089). The data of raw sequences were split by QIIME1 software (v1.8.0), then filtered and spliced using Pear software (v0.9.6). The qualified reads were clustered into operational taxonomic units (OTUs) at a similarity level of 97% by the UPARSE Pipeline method using Vsearch software (v2.7.1) to obtain the species classification information corresponding to each OTU. Then all sequences were classified into different taxonomic groups using the Ribosomal Database Project (RDP) classifier with reference to the SILVA128 database. Additionally, QIIME1 software (v1.8.0) was used to analyze the diversity indices. Finally, based on the results of species annotation and relative abundance, R software (v4.1.2) was employed to analyze and generate species composition bar charts.

### 2.5 Collection, measurement, and analysis of rumen and jejunum tissue samples

Samples from the rumen and jejunum of all slaughtered lambs were collected for optical microscopy. Tissue blocks (2 × 2 cm) from the rumen back bursa and approximately 2 cm sections from the middle jejunum were excised. After washing with physiological salt solution, samples were fixed in 4% paraformaldehyde for 24 h and then transferred to fresh 4% paraformaldehyde until analysis. According to the method reported by Han et al. (25), tissue blocks were dehydrated in graded ethanol solutions (70–100%), cleared in xylene for 1 h, infiltrated with wax at 55–60 °C, and embedded. Sections were stained with hematoxylin and eosin, and finally, the microstructure of the rumen and jejunum was examined using a panoramic slice scanner (Pannoramic Scan, 3DHISTECH, Hungary).

### 2.6 Collection, measurement, and analysis of the digestive tract

All slaughtered lambs were weighed individually before slaughter. Four stomachs (rumen, reticulum, flap stomach, and abomasum), the small intestine, and the large intestine were detached, and their contents were removed. After being cleaned, the organs were weighed individually, and the organ index was calculated: Organ index = organ weight/live weight × 100% (26).

### 2.7 Statistical analyses

All data were analyzed by a general linear model (GLM) in SAS 9.4 software. In the *in vitro* rumen fermentation, with FB dose as the fixed effect, linear and cubic effect analyses were performed using contrast statements to determine the effects of gradient doses of FB on fermentation parameters and nutrient disappearance rate. Data from the *in vivo* test were analyzed by one-way analysis of variance (ANOVA) using the GLM procedure as follows:

$Y_{\mathrm{ij}}=\mu +\alpha_{i}+\varepsilon_{\mathrm{ij}}$ ,

where *Y_ij_* is the observed value of the dependent variable, *μ* is the overall population mean, *α_i_* is the fixed treatment effect, and *ε_ij_* is the random error.

Data were presented as mean ± standard error of mean (SEM). Significant effects were represented at *p* ≤ 0.05, extremely significant effects were declared at *p* < 0.01, and tendencies were represented as 0.05 < *p* ≤ 0.10.

## 3 Results

### 3.1 *In vitro* rumen fermentation parameters

Effects of different doses of FBs on GP *in vitro* fermentation are shown in Table 2. No difference (*p* > 0.05) was observed among all groups in GP after 2 h or 4 h of fermentation in linear and quadratic effects. After 6 h to 48 h, with increasing FB dosage, GP demonstrated a quadratic pattern (*p* < 0.01), and the values in the FT2 and FT3 groups reached the peaks, which were greater (*p* < 0.05) than those in other groups, except for those after 6 h in the FT4 group. Additionally, the three parameters (a, b, and a + b) of GP were affected (quadratic, *p* < 0.01) when different doses of FBs were supplemented. The values of a, b, and a + b in FB-offered groups increased (*p* < 0.05) compared to the control group, except for the values of b and a + b in the FT5 group and a value in the FT1 group. Particularly, the values of a, b, and a + b in the FT2 and FT3 groups were greater (*p* < 0.05) than those in other groups.

**Table 2:** Effects of fermentation broth (FB) on gas production during *in vitro* rumen fermentation.

Time	Gas production/mL						SEM	*P*-value^1^	
	C	FT1	FT2	FT3	FT4	FT5		Linear	Quadratic
2 h	3.70	3.30	3.88	3.60	3.88	3.80	0.27	0.45	0.66
4 h	7.98	7.40	8.30	8.04	8.14	7.88	0.51	0.92	0.57
6 h	16.26^b^	16.46^b^	19.26^a^	19.50^a^	17.88^ab^	17.06^b^	0.63	0.92	<0.01
8 h	27.02^c^	27.08^c^	35.36^a^	36.02^a^	31.76^b^	30.76^b^	0.89	0.17	<0.01
10 h	42.42^c^	41.94^c^	61.30^a^	61.90^a^	54.46^b^	53.58^b^	1.39	<0.01	<0.01
12 h	64.40^c^	65.50^c^	90.91^a^	91.64^a^	80.52^b^	79.42^b^	1.82	<0.01	<0.01
24 h	97.62^c^	102.78^c^	140.68^a^	144.76^a^	123.58^b^	115.42^b^	2.89	0.18	<0.01
36 h	134.72^e^	146.16^d^	193.00^a^	201.94^a^	169.52^b^	157.42^c^	3.72	0.50	<0.01
48 h	155.44^e^	173.1^d^	227.42^b^	242.16^a^	197.26^c^	182.34^d^	4.79	0.85	<0.01
a^2^	13.86^c^	13.99^c^	21.72^a^	21.42^a^	18.83^b^	17.94^b^	0.76	0.03	<0.01
b^3^	218.17^c^	277.42^b^	331.05^a^	357.47^a^	281.94^b^	249.21^bc^	12.96	0.16	<0.01
c/(×10^3^%/h)^4^	0.031	0.024	0.029	0.025	0.031	0.034	0.002	0.15	0.44
a+b^5^	232.03^c^	291.41^b^	352.77^a^	378.89^a^	300.76^b^	267.15^bc^	13.22	0.21	<0.01

1 In the same row, different superscript letters indicate significant differences (*p* < 0.05), and the same superscript letters or no letters indicate no significant difference (*p* > 0.05).

2 a, gas production of fast-degradable fraction (mL).

3 b, gas production of slow-degradable fraction (mL).

4 c, rate of gas production of slow-degradable fraction (%/h).

5 a + b, theoretical gas production (mL).

Effects of FB supplements on the nutrient disappearance rate in fermentation *in vitro* are listed in Table 3. With increasing FB doses, the disappearance rates of CP and NDF followed a quadratic pattern (*p* < 0.05), with values in the FT2 and FT3 groups significantly higher (*p* < 0.05) than those in other groups. Compared to the control group, CP and NDF increased by 6.99 and 7.60% the FT2 group and by 7.36 and 8.42% in the FT3 group, respectively. No significant differences (*p* > 0.05) were observed in DM or ADF among groups.

**Table 3:** Effects of fermentation broth (FB) on nutrient disappearance rates in *in vitro* rumen fermentation.

Nutrient	Group						SEM	*P*-value^1^	
	C	FT1	FT2	FT3	FT4	FT5		Linear	Quadratic
DM	54.10	55.07	57.00	61.59	55.10	53.85	2.15	0.66	0.06
CP	64.51^c^	66.12^b^	69.02^a^	69.26^a^	67.34^b^	67.11^b^	0.40	0.10	<0.01
NDF	44.99^b^	45.82^b^	48.41^a^	48.78^a^	45.71^b^	45.23^b^	0.74	0.18	0.04
ADF	28.78	28.86	30.93	30.97	29.36	27.68	0.82	0.16	0.15

1 In the same row, different superscript letters indicate significant differences (*P* < 0.05), and the same superscript letters or no letters indicate no significant difference (*P* > 0.05).

The effects of FB supplementation on pH, NH_3_-N, MCP, and VFA levels in *in vitro* fermentation are shown in Table 4. Compared to the control group, NH_3_-N concentrations decreased (*p* < 0.05) by 6.86–9.06% across all FB groups. MCP concentrations increased significantly (*p* < 0.05) only in the FT2 and FT3 groups, by 16.22 and 18.92%, respectively. A quadratic tendency (*p* = 0.06) in MCP was observed with increasing FB doses. For VFA, all FB-supplemented groups exhibited reduced (*p* < 0.05) acetic acid concentration and acetic acid/propionic acid (A/P) ratio compared to the control group. With the increasing FB doses, the acetic acid concentration and A/P showed a linear decrease (*p* = 0.04). The concentrations of other VFAs and total VFA were not affected (*p* > 0.05) by FB supplements.

**Table 4:** Effects of fermentation broth (FB) on pH, NH_3_-N, MCP, and VFA concentrations during *in vitro* rumen fermentation.

Items	Group						SEM	*P*-value^1^	
	C	FT1	FT2	FT3	FT4	FT5		Linear	Quadratic
pH	6.27	6.20	6.30	6.30	6.23	6.28	0.03	0.29	0.38
NH_3_-N, mg/dL	17.48^a^	16.28^b^	15.98^b^	15.87^b^	16.04^b^	16.11^b^	0.68	0.48	0.11
MCP, mg/mL	0.37^b^	0.40^ab^	0.43^a^	0.44^a^	0.41^ab^	0.41^ab^	0.01	0.26	0.06
Acetic acid, mmoL/L	44.72^a^	41.96^b^	41.66^b^	40.97^b^	41.87^b^	41.42^b^	0.76	0.04	0.17
Propionic acid, mmoL/L	16.84	18.19	18.17	18.28	18.12	18.17	0.58	0.42	0.31
Isovaleric acid, mmoL/L	0.57	0.60	0.52	0.55	0.61	0.62	0.06	0.39	0.82
Butyric acid, mmoL/L	9.76	9.92	10.56	10.31	9.52	9.93	0.37	0.59	0.94
Isobutyric acid, mmoL/L	1.15	1.24	1.22	1.21	1.15	1.17	0.07	0.65	0.99
Valeric acid, mmoL/L	1.16	1.18	1.21	1.22	1.17	1.15	0.04	0.46	0.47
Total VFA, mmoL/L	61.55	60.16	59.83	59.70	59.99	59.13	1.36	0.26	0.65
Acetic acid/propanoic acid ratio	2.66^a^	2.31^b^	2.30^b^	2.24^b^	2.32^b^	2.27^b^	0.07	0.04	0.07

1 In the same row, different superscript letters indicate significant differences (*P* < 0.05), and the same superscript letters or no letters indicate no significant difference (*P* > 0.05).

Based on the results in Tables 2–4, when FBs were supplemented at 1:1000 (FT2) and 1:500 (FT3) in rumen fermentation *in vitro*, the effect was optimal, and the two doses of FB were used for the following experiment in this study.

### 3.2 Rumen fermentation parameters in fattening lambs

When FBs were supplemented in the drinking water of fattening lambs, the results on rumen fermentation parameters, including pH, NH_3_-N, MCP, and a panel of VFA, are shown in Table 5. Compared to the control group, the NH_3_-N concentration in the T2 group decreased by 15.82% (*p* = 0.04), and the MCP increased by 11.01% (*p* < 0.01). Though total VFA levels were not affected (*p* > 0.05) by FB supplements, the concentration of propionic acid demonstrated an increase (*p* < 0.05) in two FB groups compared to the control group, and the value in the T2 group was greater (*p* < 0.05) than that in the T1 group, while the A/*p* values in two FB groups were lower (*p* < 0.01) than control, with the decreases by 13.99% in the T1 and 26.24% in the T2 group. Besides, the concentration of isobutyric acid demonstrated a reduced tendency (*p* = 0.06) when lambs were offered FBs in drinking water. There was no difference (*p* > 0.05) among groups in the levels of other VFA.

**Table 5:** Rumen fermentation parameters in fattening lambs fed fermentation broth (FB).

Items	Group			SEM	*P*-value^1^
	C	T1	T2		
pH	6.67	6.83	6.60	0.24	0.63
NH_3_-N, mg/dL	9.04^a^	8.50^ab^	7.61^b^	0.47	0.04
MCP, mg/mL	4.27^b^	4.36^b^	4.74^a^	0.09	<0.01
Acetic acid, mmoL/L	44.03	45.23	45.05	3.05	0.84
Propionic acid, mmoL/L	12.84^c^	15.35^b^	17.80^a^	0.96	<0.01
Isovaleric acid, mmoL/L	0.61^c^	0.76^b^	0.95^a^	0.04	<0.01
Butyric acid, mmoL/L	9.61	9.99	10.35	0.65	0.61
Isobutyric acid, mmoL/L	1.38	1.20	1.26	0.05	0.06
Valeric acid, mmoL/L	1.05	1.24	1.31	0.10	0.11
Total VFA, mmoL/L	69.52	73.77	76.72	6.21	0.52
Acetic acid/propanoic acid ratio	3.43^a^	2.95^b^	2.53^c^	0.11	<0.01

1 In the same row, different superscript letters indicate a significant difference (*P* < 0.05), and the same superscript letters or no letters indicate no significant difference (*P* > 0.05). C - control group, T1 - FB supplement in drinking water at 1:1000; T2 - FB supplement in drinking water at 1:500.

### 3.3 Microstructure of rumen and jejunum in fattening lambs

**Figure 1:**
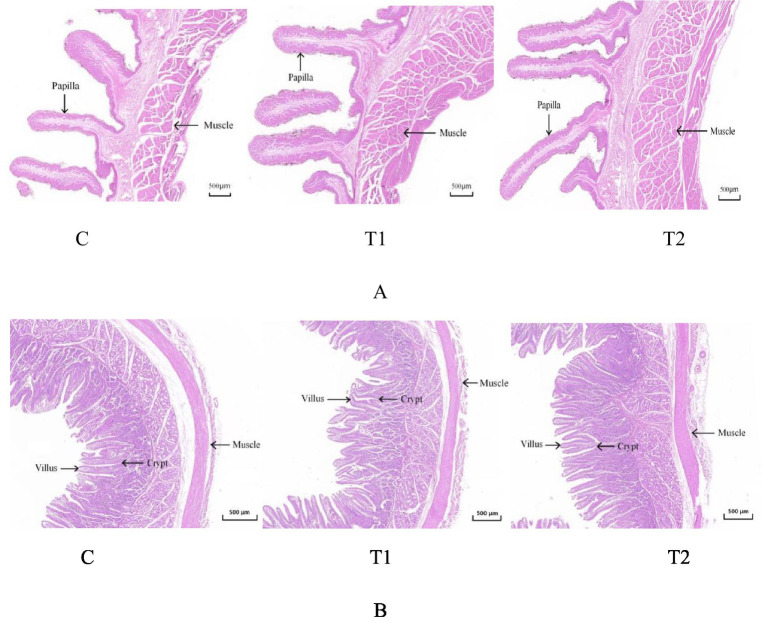
Microstructure of the rumen **(A)** and jejunum **(B)** in fattening lambs fed fermentation broth (FB). C - control group; T1 - FB supplement in drinking water at 1:1000; T2 - FB supplement in drinking water at 1:500.

Microstructural changes of the rumen and the jejunum from lambs offered FBs are shown in Figure 1 and Table 6. The four parameters in the rumen, including papilla length, mucosa thickness, muscle thickness, and papilla surface, were positively affected (*p* < 0.01) by FB supplements in drinking water. The effects observed in the T2 group were greater (*p* < 0.05) than those in the T1 group. Compared with the control group, the T1 group showed increases of 17.07, 17.28, 8.20, and 19.59% in the four parameters mentioned above, while the T2 group exhibited increases of 37.62, 38.68, 29.51, and 36.08% (Figure 1A; Table 6). Moreover, FB supplementation improved the microstructure of the jejunum, with increases (*p* < 0.05) of 6.06% in the T1 group and 19.7% in the T2 group for villus height; 17.65 and 50.0% for muscle thickness; 9.33 and 24.0% for villus surface area; and 5.16 and 15.48% for the villus height/crypt depth ratio, respectively, compared to the control group (Figure 1B; Table 6).

**Table 6:** Gastrointestinal morphology of fattening lambs fed fermentation broth (FB).

Items	Variable	Group			SEM	*P*-value^1^
		C	T1	T2		
Rumen	Papilla length/mm	2.02^c^	2.37^b^	2.78^a^	0.05	<0.01
	Papilla width/mm	0.48	0.49	0.48	0.01	0.30
	Mucosa thickness/mm	2.43^c^	2.85^b^	3.37^a^	0.04	<0.01
	Muscle thickness/mm	1.22^c^	1.32^b^	1.58^a^	0.03	<0.01
	Papilla superficial area/mm^2^	0.97^c^	1.16^b^	1.32^a^	0.02	<0.01
Jejunum	Villus height/mm	0.66^c^	0.70^b^	0.79^a^	0.01	<0.01
	Villus width/mm	0.11	0.12	0.12	0.01	0.61
	Crypt depth/mm	0.43	0.43	0.44	0.01	0.13
	Muscle thickness/mm	0.34^c^	0.40^b^	0.51^a^	0.01	<0.01
	Villus superficial area/mm^2^	0.075^c^	0.082^b^	0.093^a^	0.002	<0.01
	Villus height/Crypt depth	1.55^c^	1.63^b^	1.79^a^	0.02	<0.01

1 In the same row, different superscript letters indicate a significant difference (*P* < 0.05), and the same superscript letters or no letters indicate no significant difference (*P* > 0.05). C - control group, T1 - FB supplement in drinking water at 1:1000; T2 - FB supplement in drinking water at 1:500.

### 3.4 Organ index of the digestive tract in fattening lambs

Organ indexes of the digestive tract for fattening lambs offered FBs are shown in Table 7. There was no difference (*p* > 0.05) observed in all organ indexes among all groups.

**Table 7:** Organ index of the digestive tract for fattening lambs fed fermentation broth (FB).

Organ	Group			SEM	*P*-value
	C	T1	T2		
Rumen	14.11	14.63	15.06	0.59	0.34
Reticulum	3.26	3.12	3.34	0.29	0.76
Omasum	3.75	3.81	3.71	0.23	0.91
Abomasum	4.94	4.69	4.77	0.30	0.72
Small intestine	17.77	17.58	17.90	0.54	0.84
Large intestine	11.44	11.15	11.35	0.36	0.73

C - control group; T1 - FB supplement in drinking water at 1:1000; T2 - FB supplement in drinking water at 1:500.

### 3.5 Microbial diversity in the rumen and jejunum in fattening lambs

**Figure 2:**
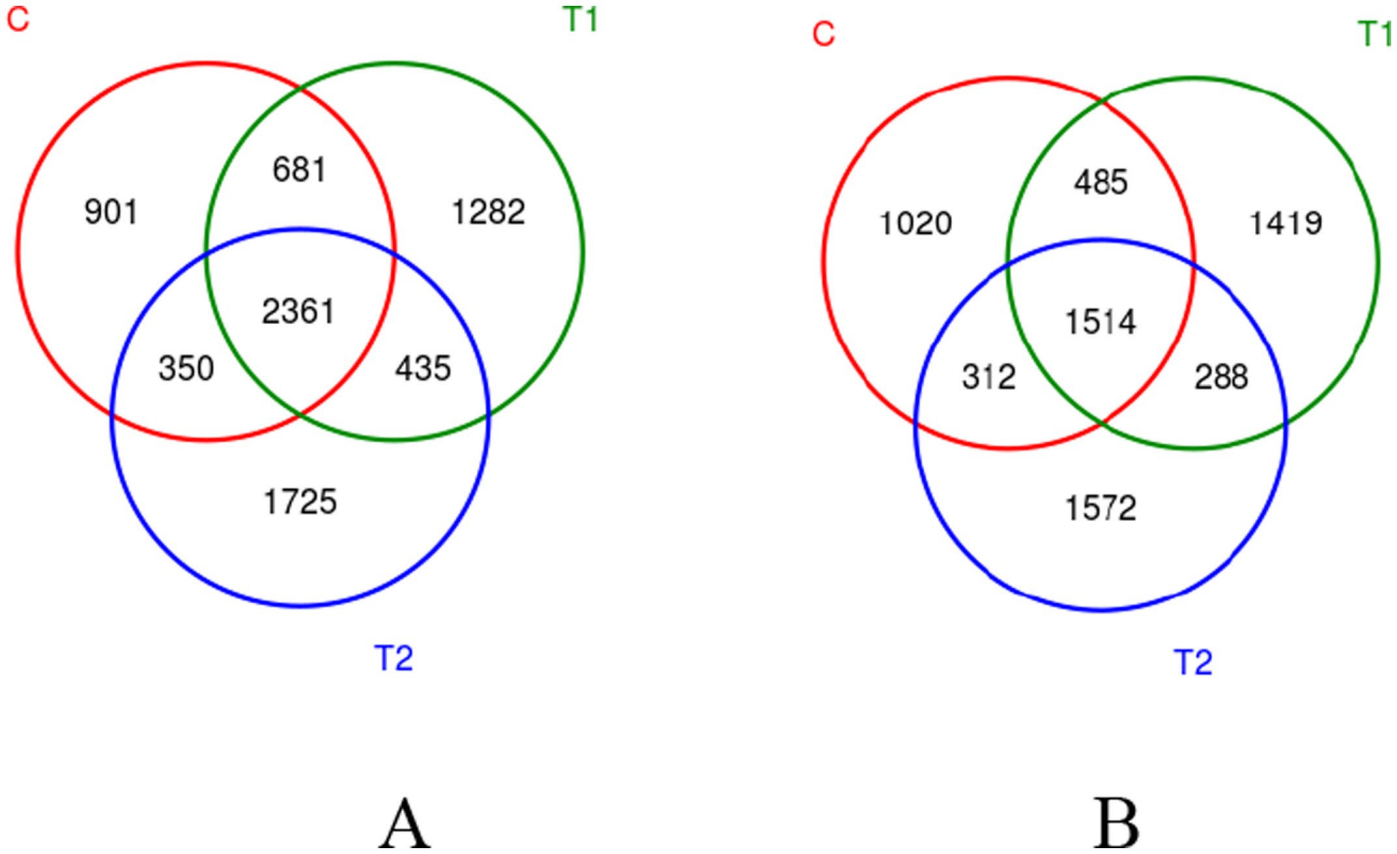
Venn diagram showing OTU distribution in bacterial communities in the rumen **(A)** and jejunum **(B)**. The overlapping area indicates shared OTUs, while the non-overlapping area shows unique OTUs. C - control group, T1 - FB supplement in drinking water at 1:1000, T2 - FB supplement in drinking water at 1:500.

The FB supplements improved the diversity of the bacterial community in the rumen and jejunum, as shown in the Venn diagram in Figure 2. In both the rumen and jejunum, the OTU numbers in the two FB groups were greater than in the control. The common OTU in all groups were observed with 2,361 in rumen and 1,514 in jejunum, while unique OTU in the control group, T1, and T2 groups were 901, 1,282, and 1725 in rumen, respectively, and 1,020, 1,419, and 1,572 in jejunum, respectively.

**Figure 3:**
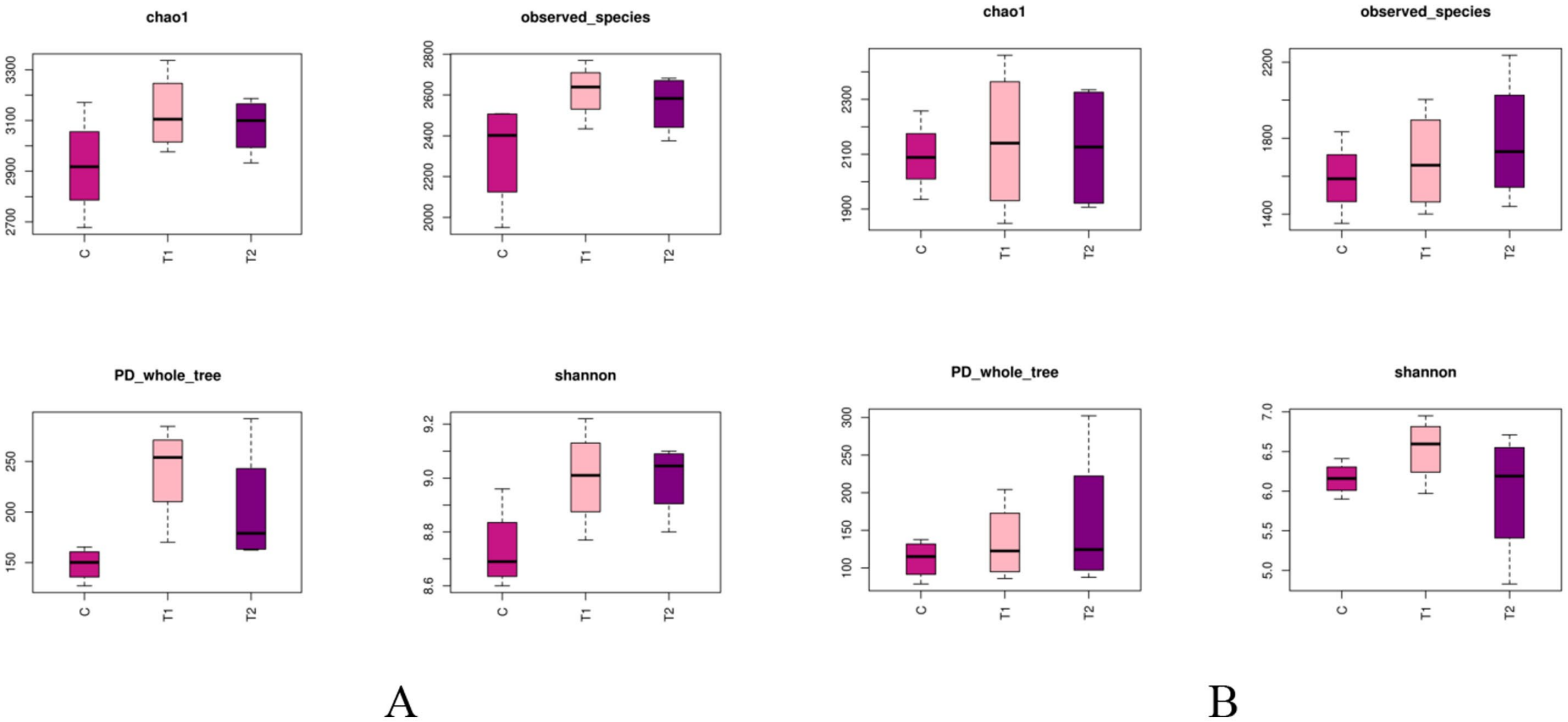
Alpha-diversity indexes of bacterial communities in the rumen **(A)** and jejunum **(B)** of fattening lambs fed fermentation broth (FB). C - control group, T1 - FB supplement in drinking water at 1:1000; T2 - FB supplement in drinking water at 1:500. Differences in alpha-diversity metrics in rumen and jejunum: (1) Rumen: Chao1 (*p* = 0.21), Observed_species (*p* = 0.11), PD_whole_tree (*p* = 0.06), and Shannon (*p* = 0.07). (2) Jejunum: Chao1 (*p* = 0.94), Observed_species (*p* = 0.62), PD_whole_tree (*p* = 0.60), and Shannon (*p* = 0.39).

**Figure 4:**
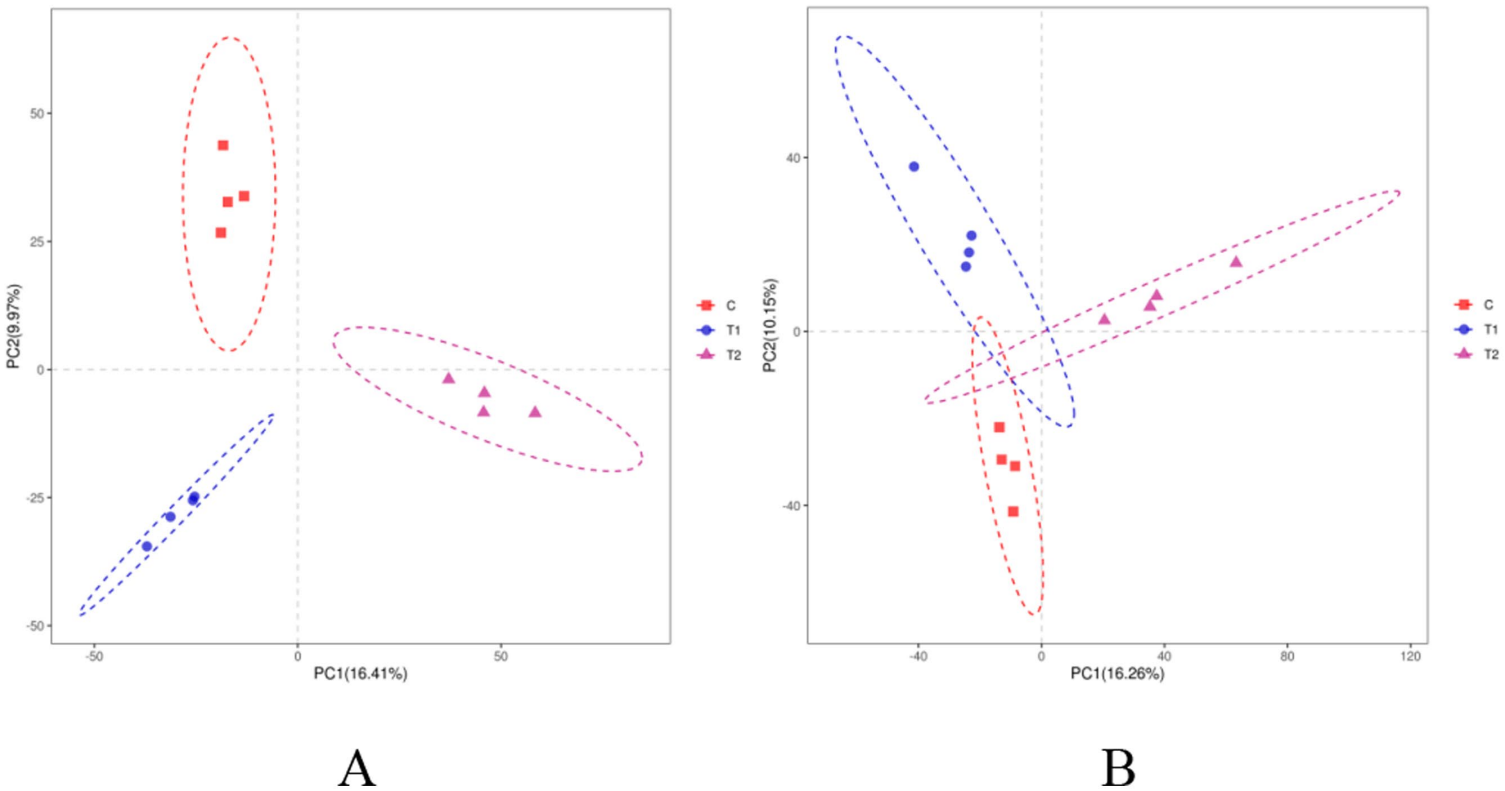
Beta-diversity analysis of bacterial communities in the rumen **(A)** and jejunum **(B)** of fattening lambs offered fermentation broth (FB), based on weighted UniFrac distance. C - control group, T1 - FB supplement in drinking water at 1:1000, T2 - FB supplement in drinking water at 1:500.

The alpha-diversity indexes of bacterial communities in the rumen and jejunum are listed in Figure 3. Two indices of PD_whole_tree (*p* = 0.06) and Shannon (*p* = 0.07) for rumen showed an increased trend when doses of FB were provided to lambs (Figure 3A), suggesting that FB improved the diversity of the rumen bacterial community. However, no difference (*p* > 0.05) was found among all groups in all alpha-diversity indexes for the jejunum (Figure 3B). Additionally, from the beta-diversity analysis (Figures 4A,B), no overlap was observed among all groups for rumen, which suggested that there was a great difference among groups in the bacterial community. However, there was a small overlap among groups for the jejunum, suggesting that the microbial composition presented some similarity among groups. ANOSIM analysis also showed that the bacterial communities among groups demonstrated the differences in rumen (*R* = 0.0.7986, *p* = 0.001) and jejunum (*R* = 0.6713, *p* = 0.001) (Table 8).

**Table 8:** ANOSIM analysis of bacterial communities in the rumen and jejunum of fattening lambs fed fermentation broth (FB).

Group	R statistic		*P*-value	
	Rumen	Jejunum	Rumen	Jejunum
C-T1	0.6042	0.5729	0.025	0.069
C-T2	0.9167	0.7500	0.029	0.027
T1-T2	1.0000	0.7500	0.029	0.021
All	0.7986	0.6713	0.001	0.001

C - control group, T1 - FB supplement in drinking water at 1:1000; T2 - FB supplement in drinking water at 1:500.

### 3.6 Microbial composition in rumen and jejunum in fattening lambs

**Figure 5:**
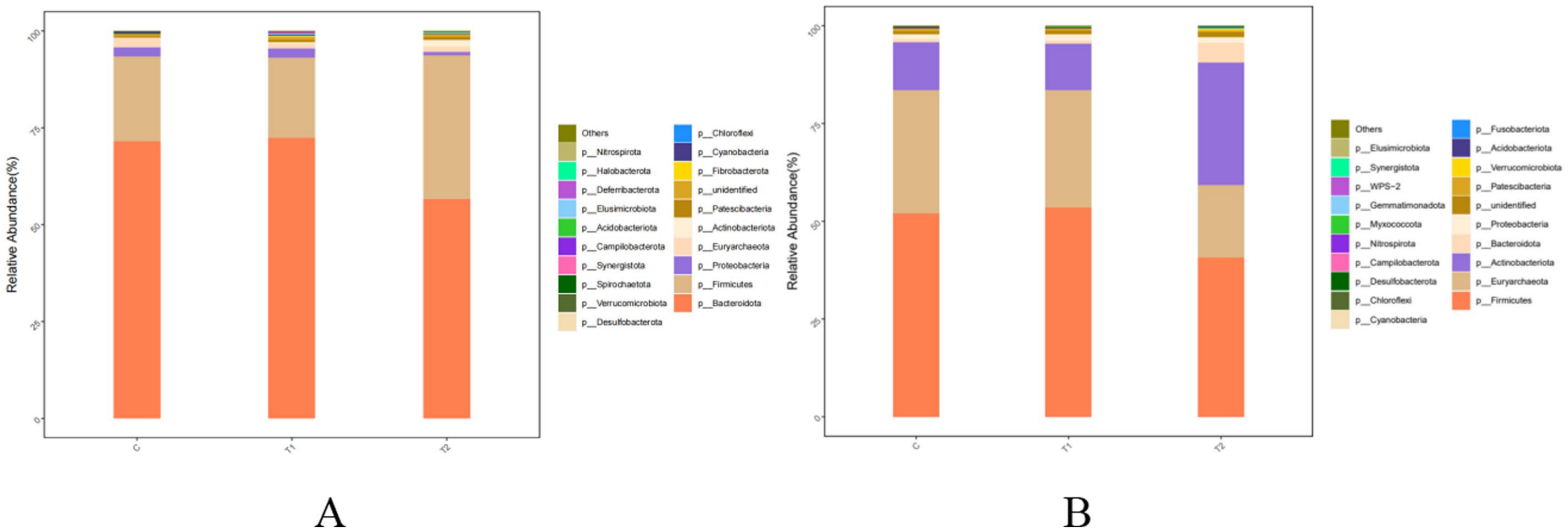
Composition and relative abundance of bacterial communities in the rumen **(A)** and jejunum **(B)** of fattening lambs fed fermentation broth (FB) at the phylum level. C - control group, T1 - FB supplement in drinking water at 1:1000; T2 - FB supplement in drinking water at 1:500.

The effects of FBs on species compositions at the phylum level of bacterial community in rumen and jejunum are shown in Figure 5 and Table 9. *Bacteroidota* and *Firmicutes* were observed as dominant bacteria in the rumen. In the T2 group, the relative abundance of *Firmicutes* increased by 70.75% (*p* < 0.01), while the abundance of *Bacteroidota* decreased by 20.97% (*p* < 0.01), compared to the control group. In contrast, no significant differences (*p* > 0.05) in the abundances of either *Bacteroidota* or *Firmicutes* were observed between the T1 and the control groups (Figure 5A; Table 9). In the jejunum, the dominant bacterial phyla were *Firmicutes*, *Euryarchaeota*, and *Actinobacteota*. The relative abundances of these three phyla in the T1 group showed no difference (*p* > 0.05) between the T1 and the control group. However, compared to the control group, the abundances of *Firmicutes* (*p* = 0.01) and *Euryarchaeota* (*p* = 0.02) in the T2 group decreased by 21.59 and 41.62%, respectively, while *Actinobacteota* abundance increased by 1.59 times in the T2 group (Figure 5B; Table 9).

**Table 9:** Composition and relative abundance of bacterial communities in the rumen and jejunum of fattening lambs fed fermentation broth (FB) at the phylum level (relative abundance > 0.1%).

Items	Group			SEM	*P*-value^1^
	C	T1	T2		
Rumen					
Bacteroidota	71.52^a^	72.47^a^	56.52^b^	2.95	<0.01
Firmicutes	21.81^b^	20.63^b^	37.24^a^	3.11	<0.01
Proteobacteria	2.51	2.41	0.89	0.88	0.17
Euryarchaeota	2.32	1.22	1.35	1.28	0.66
Actinobacteriota	0.21^b^	0.40^b^	1.78^a^	0.22	<0.01
Patescibacteria	0.60	0.63	0.68	0.23	0.94
Fibrobacterota	0.15^b^	0.27^a^	0.15^b^	0.05	0.04
Cyanobacteria	0.30	0.14	0.12	0.19	0.61
Jejunum					
Firmicutes	52.05^a^	53.59^a^	40.81^b^	3.67	0.01
Euryarchaeota	31.62^a^	29.99^a^	18.46^b^	3.87	0.02
Actinobacteriota	12.06^b^	11.91^b^	31.28^a^	5.31	0.01
Bacteroidota	0.92^b^	0.72^b^	5.12^a^	0.78	<0.01
Proteobacteria	1.30	1.73	1.45	1.01	0.91
Patescibacteria	0.72	0.65	0.51	0.21	0.58
Acidobacteriota	0.29	0.15	0.11	0.17	0.54

1 In the same row, different superscript letters indicate a significant difference (*P* < 0.05), and the same superscript letters or no letters indicate no significant difference (*P* > 0.05). C - control group, T1 - FB supplement in drinking water at 1:1000; T2 - FB supplement in drinking water at 1:500.

**Figure 6:**
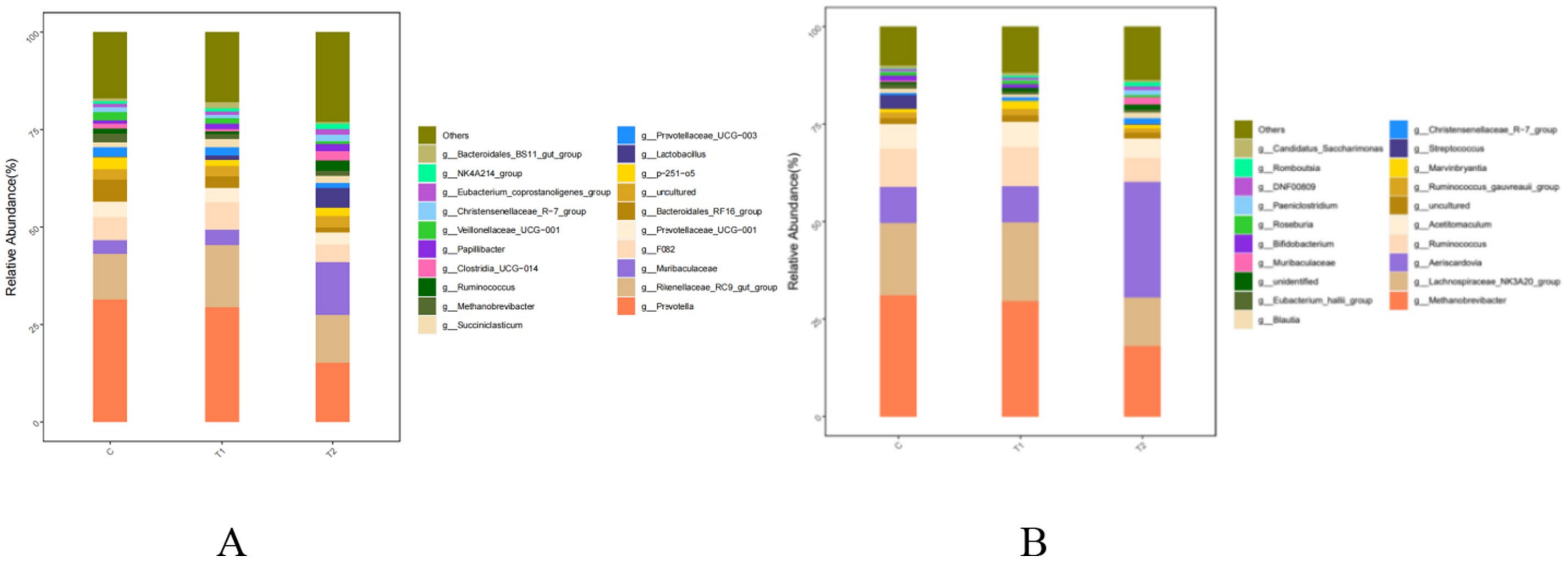
Composition and relative abundance of bacterial communities in the rumen **(A)** and jejunum **(B)** of fattening lambs fed fermentation broth (FB) at the genus level. C - control group, T1 - FB supplement in drinking water at 1:1000; T2 - FB supplement in drinking water at 1:500.

Bacterial compositions at the genus level in the rumen and jejunum were affected by FB supplements, and the results are shown in Figure 6 and Table 10. In rumen bacteria, compared to the control group, the abundance of *Prevotella*, as a main dominant genus, demonstrated a decrease (*p* < 0.01) in the T2 group, while the *Rikenellaceae_RC9_gut_group*, as the second dominant genus, was increased (*p* = 0.05) by FB supplements, exhibiting the increases by 35.46% in the T1 and 4.77% in the T2 groups. Meanwhile, the abundance of *Muribaculaceae* (non-dominant genus) in the T2 group was 2.94 times greater (*p* < 0.01) than that in the control group (Figure 6A; Table 10). Additionally, two dominant bacteria in the jejunum, including *Methanobrevibacter* and *Lachnospiraceae_NK3A20_group*, were also altered by FB supplements, revealing a decline of 41.68% (*p* = 0.02) and 32.60% (*p* = 0.01) in the T2 group, respectively, compared to the control group. Besides, the abundance of *Aeriscardovia* in the jejunum in the T2 group was 2.16 times greater (*p* = 0.01) than in the control, while the *Ruminococcus* (*p* = 0.02) and *Acetitomaculum* (*p* = 0.01) in the T2 group demonstrated a decrease (Figure 6B; Table 10).

**Table 10:** Composition and relative abundance of bacterial communities in the rumen and jejunum of fattening lambs fed fermentation broth (FB) at the genus level (relative abundance > 1%).

Items	Group			SEM	*P*-value^1^
	C	T1	T2		
Rumen					
Prevotella	31.50^a^	29.45^a^	15.20^b^	2.96	<0.01
Rikenellaceae_RC9_gut_group	11.73^b^	15.89^a^	12.29^b^	1.57	0.05
Muribaculaceae	3.45^b^	4.07^b^	13.58^a^	1.42	<0.01
F082	5.84	6.97	4.53	1.14	0.16
Prevotellaceae_UCG-001	4.06	3.68	3.04	0.61	0.29
Bacteroidales_RF16_group	5.61^a^	3.04^b^	1.24^c^	0.52	<0.01
P-251-o5	3.00	1.67	1.98	0.69	0.18
Prevotellaceae_UCG-003	2.44^a^	2.11^a^	1.28^b^	0.32	0.02
Succiniclasticum	1.19	2.21	1.78	0.50	0.17
Methanobrevibacter	2.32	1.22	1.31	1.28	0.65
Jejunum					
Methanobrevibacter	31.12^a^	29.54^a^	18.15^b^	3.90	0.02
Lachnospiraceae_NK3A20_group	18.56^a^	20.31^a^	12.51^b^	2.01	0.01
Aeriscardovia	9.35^b^	9.32^b^	29.54^a^	5.97	0.01
Ruminococcus	9.72^a^	9.99^a^	6.10^b^	1.23	0.02
Acetitomaculum	6.33^a^	6.49^a^	4.98^b^	0.42	0.01
Marvinbryantia	1.06^b^	2.00^a^	1.08^b^	0.34	0.04

1 In the same row, different superscript letters indicate a significant difference (*P* < 0.05), and the same superscript letters or no letters indicate no significant difference (*P* > 0.05). C - control group, T1 - FB supplement in drinking water at 1:1000; T2 - FB supplement in drinking water at 1:500.

## 4 Discussion

Fermentation parameters involving GP and nutrient disappearance rates were investigated in this study when the FB was supplemented to artificial rumen fluid in the experiment of *in vitro* fermentation. Generally, GP plays an important role in rumen fermentation, and it is positively correlated with nutrient degradation rates (27). Our study shows that the 48-h GP of fast-and slow-degradable fractions, as well as theoretical GP, increased significantly with FB supplementation at doses ranging from 1:1000 to 1:250. Notably, the 1:1000 and 1:500 doses produced the highest GP among all FB levels, suggesting that FB may promote the degradation of nutrients by microorganisms. This finding is consistent with the higher disappearance rates of CP and NDF observed at these two doses in this study. However, the GP of the slow-degradable fraction and the theoretical GP were not affected by the highest dose of FB (1:250). The reports from Bachmann et al. (28) indicated that low or high levels of prebiotics might reduce GP of *in vitro* fermentation, and our present results also suggest that only appropriate doses of FBs could promote the degradation of nutrients.

The effects of FBs on fermentation parameters (pH, NH_3_-N, and MCP) in both *in vitro* and *in vivo* tests were also examined in this study. The NH_3_-N is a main nitrogen source of MCP synthesis for ruminants and reflects the balance between synthesis and degradation of proteins in the substrate. Moreover, MCP in the rumen also affects the utilization of ruminal NH_3_-N by microorganisms, and more MCP production contributes to the enhancement of the utilization efficiency of nitrogen (29), which is generally related to microbial diversity in the rumen. Our data in the present study demonstrate a significant decrease in NH_3_-N concentration and a significant increase in MCP concentration when the FB of 1:500 was added to drinking water, which could be explained by the changes in microbial diversity in the rumen or jejunum. From the Venn diagram and alpha-diversity index, whether in rumen or jejunum, the FB supplements of 1:500 to 1:1000 significantly improved the diversity of the bacterial community, exhibiting that the unique OTU number increased by 42.3 to 91.5% in rumen and 39.1 to 54.1% in jejunum. The changes of bacterial community could promote the transformation of NH_3_-N to MCP and meanwhile consume more NH_3_-N, which is in agreement with Ghorbani et al. (30). Also, our present results of NH_3_-N and MCP in the *in vivo* experiment are consistent with those in the fermentation experiment *in vitro*. In an *in vitro* experiment, different levels of FBs reduced the NH_3_-N content in artificial rumen fluid. Particularly for the FB doses between 1:500 and 1:1000, the NH_3_-N decreased by 8.58 to 9.21%, while the MCP increased by 16.22 to 18.92% when compared to the control group. These results suggest that probiotics or other unknown active ingredients in FB may have some positive effects. Previous studies on probiotics (e.g., Bacillus) also showed a decrease in NH_3_-N content and an increase in MCP in rumen or the intestine (31), with which our present results are in agreement.

A panel of VFA parameters in the rumen of lambs offered FB supplements was also measured in this study. The content of isobutyric acid was increased by the FB supplements of 1:500 or 1:1000 in drinking water in the *in vivo* test. Published literature has demonstrated that isobutyric acid might accelerate the reproduction of rumen cellulolytic bacteria, resulting in the enhancement of nutrient digestion, particularly cellulose (32, 33). In this case, the NDF degradation rate was elevated by 1:500 or 1:1000 FB offered in drinking water, which suggests that FB would prefer to promote the degradation of cellulose to some degree. However, the isobutyric acid content did not show differences among all groups in the *in vitro* test. This is due to different experimental conditions between the *in vivo* and *in vitro* tests. Similarly, the propionic acid content also increased in two FB-offered groups in the *in vivo* test, though the content demonstrated no differences among all groups in the *in vivo* test. Propionic acid, as an important VFA in the rumen, has been proven in recent studies to have its augmentation promote more energy to translate into body weight in fattening animals, providing greater benefits for weight gain (34). Moreover, in this experiment, whether *in vivo* or *in vitro*, the A/P ratio in rumen liquid demonstrates a decrease due to the addition of FB. However, the greater reduction magnitudes for the 1:500 and 1:1000 groups were observed in the *in vivo* test compared to the *in vitro* test (26.24% vs. 15.79% for 1:500; 13.99% vs. 13.53% for 1:1000). These results suggest a potential shift in rumen fermentation pattern toward propionic acid-producing pathways. This pattern is usually deemed a highly efficient mode of energy utilization in animals or microorganisms. The decrease of A/P here may be explained by the fact that our FB contains many bioactive components such as polysaccharides, saponins, and flavonoids, which could promote the digestion and absorption of nutrients and improve the energy utilization. Early studies have demonstrated that dietary polysaccharides, saponins, or flavones supplements improved the growth performance involving weight gain and feed conversion rate in animals (35–37). Moreover, the bioactive substances would stimulate the development of the rumen and alter its microstructure to help digestion (38). It is well known that rumen development is very important for ruminants, and a well-developed rumen contributes to feed conversion and energy utilization (39). In this study, under a microscope, the rumen microstructure was influenced by FB supplements, and the increases in papilla length, mucosa thickness, muscle thickness, and papilla surface were observed. In general, papilla length and its surface are important indices to evaluate rumen morphology. Particularly, the papilla surface may be directly associated with the chance of chyme touching the rumen, which suggests that more papilla surface would facilitate nutrient absorption in the rumen (40). In addition, the jejunum, as a part of the small intestine, has strong decomposing and absorbing functions on nutrients (41). In this experiment, FB supplementation promoted jejunum development in lambs. Microstructural parameters, including villus height, muscle thickness, villus surface, and the villus height/crypt depth ratio, were improved, thereby increasing the contact area between nutrients and the jejunum and enhancing nutrient absorption (42). Previous studies suggested that the development of the gastrointestinal tract was also associated with feed additives, gastrointestinal microecology, and diet composition (43). In our experiment, the FB contained some saponin, which is extracted mainly from alfalfa. Saponin has been reported to promote the reproduction of beneficial bacteria in the gastrointestinal tract (44). This may be due to its ability to modulate the microenvironment suitable for gastrointestinal microorganisms, thereby promoting the development of the rumen and jejunum (45).

The majority of published studies have reported that maintaining a balance of gastrointestinal microorganisms is helpful to protect animal health and improve its digestibility (46–48). The FB in this study may enrich a variety of bioactive ingredients (such as alfalfa flavonoid, alfalfa saponin, and astragalus polysaccharide), as well as some probiotics such as Lactobacillus. Many studies on probiotics improving microbial communities have been reported in the past few decades (49, 50). It is generally believed that probiotics enter the gastrointestinal tract via dietary mixture and then compete with, inhibit, and coexist with inherent gastrointestinal-colonizing bacteria, eventually forming a stable and healthy gastrointestinal environment (51). In this study, three analysis methods of data, including Venn diagram, alpha-diversity, and beta-diversity, were used to analyze the effects of FBs on the microbial community in the gastrointestinal tract of fattening lambs. The FB supplements in drinking water significantly improve the diversity of microbial communities in the rumen and jejunum, particularly in the rumen. As mentioned above, the unique OTU in the rumen increased by 91.5% when the FB of 1:500 was provided in drinking water for lambs, and the PD_whole_tree and Shannon index in the rumen had an increased tendency for FB groups compared with the control group. The reasons may be related to the flavonoids contained in the FBs. Flavonoids have some positive effects on microbial communities in the digestive tracts, e.g., improving microbial diversity, increasing beneficial bacteria (e.g., *Bifidobacteria* and *Lactobacillus*) count, and inhibiting the reproduction of harmful bacteria by destroying the cell membrane and slowing down their metabolism (52, 53). Similar studies also showed that dietary flavonoid supplements could effectively inhibit the reproduction of *Escherichia coli* in the gut and enhance the abundance of *Bifidobacteria* (54). Additionally, the microbial composition and abundance of FB-offered lambs at the phylum and genus levels were also investigated in this study. Two dominant bacterial phyla in the rumen were found, including Bacteroidota and Firmicutes, which agrees with previous results (55, 56). Bacteroidota plays an important role in decomposing polysaccharides, while Firmicutes can increase the activity of gene-coding enzymes, promote rumen development, and improve the absorption capacity of starch and cellulose (57). The results of this experiment show that FB supplementation at 1:500 in drinking water increased the abundance of phylum Firmicutes in the rumen by 70.75% and decreased the abundance of phylum Bacteroidota compared with the control group. However, two rumen bacteria subordinate to the phylum Bacteroidota, including *Rikenellaceae_RC9_gut_group* (dominant bacterial genus) and *Muribaculaceae* (non-dominant bacterial genus), demonstrated significant increases in abundance, with the *Muribaculaceae* abundance in the 1:500 group being 2.9 times greater than the control. The study by Berry et al. (58) indicated that *Rikenellaceae_RC9_gut_group* mainly exerts the effects in promoting the digestion and absorption of carbohydrates, which are known as a main energy source in ruminants and can be converted into VFA to provide more energy. Though the *Muribaculaceae* is not the dominant bacterium in the rumen, it has some functional diversity in decomposing complex carbohydrates. However, little relevant information is available (59). Thus, the FB supplements in drinking water in our study could promote the degradation of carbohydrates in the rumen under the synergistic action of the bacterial population. From analysis of microbial composition in the jejunum, Firmicutes, as the main dominant bacterial phylum, were not affected in abundance by FB supplements. Euryarchaeota, the second dominant bacterial phylum, is traditionally called the methanogenic bacteria because it is mainly involved in methane production. The abundances of phylum Euryarchaeota and *Methanobrevibacter* (the main dominant bacterial genus) subordinate to phylum Euryarchaeota were lowered by the 1:500 addition of FB. Results from Djemai et al. (60) indicated that the genus *Methanobrevibacter* mainly participates in methane production under anaerobic conditions. The decrease in the abundance of methanogenic bacteria in our data may be due to the fact that flavonoids from alfalfa, *Platycladus orientalis*, and *Astragali Radix* in the FB inhibit the activity of methanogenic bacteria (61), which would lead to the reduction of methane production and improve the energy utilization for ruminants. A recent study also suggested that saponin in alfalfa could reduce the methane-producing bacteria count or their activity, thereby reducing methane concentration (62). In addition, the addition of 1:500 FB increased the phylum Actinomycetes abundance in the jejunum. Evidence has demonstrated that Actinomycetes take part in the degradation of organic matter and nutrient absorption and reduce the occurrence of diarrhea (63). The *Aeriscardovia* abundance in the jejunum, belonging to the phylum Actinomycetes, increased when 1:500 FB was provided to lambs by the addition to drinking water. This genus, *Aeriscardovia,* which is subordinate to the family Bifidobacteriaceae, has been proven to have these positive functions, e.g., inhibiting harmful bacteria growth, maintaining a balanced microenvironment in gastrointestinal tracts, and improving immunity (64). The reason for this change in our study is associated with polysaccharides in FB. A published study from Shang et al. (65) has demonstrated that this bioactive ingredient can promote the growth of *Bifidobacteria*. Meanwhile, the *Lactobacillus* in FB may play an important role in improving the microbial structure of the rumen and jejunum. It can not only effectively inhibit the colonization and proliferation of harmful bacteria by competing with intestinal pathogenic bacteria for adhesion sites and nutritional resources, thereby maintaining intestinal microbiota homeostasis, but also metabolically produce new bioactive substances (such as enzymes, vitamins, bacitracin). These substances can inhibit the reproduction of intestinal pathogenic bacteria, thus enhancing the efficiency of intestinal digestion and absorption of nutrients (66, 67). Therefore, from all results of our two experiments (*in vivo* and *in vitro*), the positive effects involving digestive tract development and microbial community caused by FB supplements would be comprehensive responses to FB products.

## 5 Conclusion

The data from *in vitro* and *in vivo* experiments suggest that FB supplementation can modulate rumen fermentation in fattening lambs. In *in vitro* rumen fermentation, FB at ratios of 1:1000 or 1:500 yielded optimal GP and nutrient disappearance rates. Moreover, supplementation at 1:500 in drinking water promoted gastrointestinal development and improved microbial community structure, thereby enhancing the digestion and absorption of nutrients.

## Data Availability

The datasets presented in this study can be found in online repositories: SRA database in the National Center for Biotechnology Information (NCBI, www.ncbi.nlm.nih.gov, PRJNA1047089.

## References

[1] Roy BC Ray BC. Potentiality of *Saccharomyces cerevisiae* in replacing antibiotic growth promoters on growth, gut microbiology, histology, and serum antibody titers of commercial broilers. J Appl Poult Res. (2023) 32:100352. doi: 10.1016/J.JAPR.2023.100352

[2] Meng WS Zou Q Xiao Y Ma W Zhang J Wang T. Growth performance and cecal microbiota of broiler chicks as affected by drinking water disinfection and/or herbal extract blend supplementation. Poult Sci. (2023) 102:102707. doi: 10.1016/J.PSJ.2023.102707, PMID: 37216884 PMC10209021

[3] Tu WL Zhang WY Wang HY Zhang YY Huang J Li B. Effects of Chinese herbal feed additives on the sperm quality and reproductive capacity in breeding boars. Front. Vet. Sci. (2023) 10:1231833. doi: 10.3389/FVETS.2023.1231833, PMID: 37565082 PMC10410075

[4] C C M C Z G Z M G T A DB. Effect of pre-and post-weaning dietary supplementation with Digestarom® herbal formulation on rabbit carcass traits and meat quality. Meat Sci. (2016) 118:89–95. doi: 10.1016/j.meatsci.2016.03.022 27062101

[5] Saleh AA Soliman MM Yousef MF Eweedah NM El-Sawy HB Shukry M. Effects of herbal supplements on milk production quality and specific blood parameters in heat-stressed early lactating cows. Front. Vet. Sci. (2023) 10:1180539. doi: 10.3389/FVETS.2023.1180539, PMID: 37332736 PMC10274320

[6] Cunha MGD Alba DF Leal KW Marcon H Milarch CF Baldissera MD. Microencapsulated herbal components in the diet of Lacaune ewes: impacts on physiology and milk production and quality. An Acad Bras Cienc. (2023) 95:e20201805. doi: 10.1590/0001-3765202320201805, PMID: 37075373

[7] Li H Liu Y Wei L Lin Q Zhang Z. Effects of feeding fermented *Medicago Sativa* (plus soybean and Ddgs) on growth performance, blood profiles, gut health, and carcass characteristics of Lande (meat) geese. Front Physiol. (2022) 13:902802. doi: 10.3389/fphys.2022.902802, PMID: 35910570 PMC9326169

[8] Dong W Fan Z Li P Liu J Sun G Peng N. Optimizing the scale-up production of fermented astragalus and its benefits to the performance and egg quality of laying hens. Front Microbiol. (2023) 14:1165644. doi: 10.3389/fmicb.2023.1165644, PMID: 37180273 PMC10169715

[9] Rho YJ Wey D Zhu ZL Kiarie E Moran K Heugten EV. Growth performance, gastrointestinal and digestibility responses in growing pigs when fed corn–soybean meal-based diets with corn DDGS treated with fiber degrading enzymes with or without liquid fermentation. J Anim Sci. (2018) 96:5188–97. doi: 10.1093/jas/sky369 30239817 PMC6276586

[10] Hoque J Deka RJ Sarma NK Ahmed HF Laskar SK. Growth response of Assam local goats under intensive farming. Agricult. Sci. Dig. Res. J. (2021) 41:256–9. doi: 10.18805/AG.D-5173

[11] Du Z Risu N Gentu G Jia Y Cai Y. Growth performance, apparent digestibility, and N balance in Mongolian lambs and hoggs fed diets supplemented with a Chinese traditional herbal medicine complex. Anim Sci J. (2018) 89:1451–8. doi: 10.1111/asj.13081, PMID: 30009556

[12] Jia P Cui K Ma T Wan F Wang W Yang D. Influence of dietary supplementation with Bacillus licheniformis and *Saccharomyces cerevisiae* as alternatives to monensin on growth performance, antioxidant, immunity, ruminal fermentation and microbial diversity of fattening lambs. Sci Rep. (2018) 8:16712. doi: 10.1038/s41598-018-35081-4, PMID: 30420720 PMC6232095

[13] Abdul-Sada SAA Abd Al-Abbas WNA Al-Gharawi JK. Effect of methods of adding red ginseng on some productive traits of laying hens. IOP Conf. Ser. Earth Environ. Sci. (2023) 1262:072030. doi: 10.1088/1755-1315/1262/7/072030

[14] Olstorpe M Lyberg K Lindberg JE Schnürer J Passoth V. Population diversity of yeasts and lactic acid bacteria in pig feed fermented with whey, wet wheat distillers' grains, or water at different temperatures. Appl Environ Microbiol. (2008) 74:1696–703. doi: 10.1128/AEM.02231-07, PMID: 18223110 PMC2268323

[15] Menke KH Raab L Salewski A Steingass H Fritz D Schneider W. The estimation of the digestibility and metabolizable energy content of ruminant feedingstuffs from the gas production when they are incubated with rumen liquor in vitro. J Agric Sci. (1979) 93:217–22. doi: 10.1017/S0021859600086305

[16] Orskov ER McDonald I. The estimation of protein degradability in the rumen from incubation measurements weighted according to rate of passage. J Agric Sci. (1979) 92:499–503. doi: 10.1017/S0021859600063048

[17] Ullah MA Kim KH Szulejko JE Cho J. The gas chromatographic determination of volatile fatty acids in wastewater samples: evaluation of experimental biases in direct injection method against thermal desorption method. Anal Chim Acta. (2014) 820:159–67. doi: 10.1016/j.aca.2014.02.012 24745750

[18] Chaney AL Marbach EP. Modified reagents for determination of urea and ammonia. Clin Chem. (1962) 8:130–2. doi: 10.1016/0009-8981(62)90080-3 13878063

[19] Cone JW Van Gelder AH Driehuis F. Description of gas production profiles with a three-phasic model. Anim Feed Sci Technol. (1997) 66:31–45. doi: 10.1016/S0377-8401(96)01147-9

[20] Bodas R López S Fernandez M García-González R Rodríguez AB Wallace RJ. In vitro screening of the potential of numerous plant species as antimethanogenic feed additives for ruminants. Anim Feed Sci Technol. (2008) 145:245–58. doi: 10.1016/j.anifeedsci.2007.04.015

[21] Lee MH. Official methods of analysis of AOAC international. Trends Food Sci Technol. (1995) 6:382. doi: 10.1016/0924-2244(95)90022-5

[22] Soest PV. Use of detergents in analysis of fibrous feeds. III. Study of effects of heating and drying on yield of fiber and lignin in forages. J Assoc Off Agric Chem. (1965) 48:785–90. doi: 10.1093/jaoac/48.4.785

[23] Zhao S Li X Gao Y Che D Zhao J Zhang H. Effects of drinking water temperature on growth performance, slaughter performance, digestibility and physiology of fattening lambs in winter. Chin. J. Anim. Nutr. (2021) 33:5749–59. doi: 10.3969/j.issn.1006-267x.2021.10.034

[24] NY 5027. Pollution-free food: Drinking water quality of livestock and poultry. Beijing: Ministry of Agriculture (2008).

[25] Han H Zhang L Shang Y Wang M Phillips CJ Wang Y. Replacement of maize silage and soyabean meal with mulberry silage in the diet of hu lambs on growth, gastrointestinal tissue morphology, rumen fermentation parameters and microbial diversity. Animals. (2022) 12:1406. doi: 10.3390/ani12111406, PMID: 35681869 PMC9179289

[26] Zhang JP Wei QX Li QL Liu RF Tang LQ Song YX. Effects of hybrid *Broussonetia papyrifera* silage on growth performance, visceral organs, blood biochemical indices, antioxidant indices, and carcass traits in dairy goats. Anim Feed Sci Technol. (2022) 292:115435. doi: 10.1016/J.ANIFEEDSCI.2022.115435

[27] Seyedin SMV Ghavipanje N Mojtahedi M Farhangfar SH Vargas-Bello-Pérez E. Inclusion of *Berberis vulgaris* leaf in the diet of fattening lambs: effects on performance, nutrient intake, rumen fermentation, and carcass traits. J Anim Sci. (2023) 101:skad131. doi: 10.1093/jas/skad131, PMID: 37105718 PMC10205459

[28] Bachmann M Glatter M Bochnia M Wensch-Dorendorf M Greef JM Breves G. In vitro gas production from batch cultures of stomach and hindgut digesta of horses adapted to a prebiotic dose of fructooligosaccharides and inulin. J Equine Vet Sci. (2020) 90:103020. doi: 10.1016/j.jevs.2020.103020, PMID: 32534784

[29] Sheikh GG Ganai AM Ahmad Sheikh A Mir DM. Rumen microflora, fermentation pattern and microbial enzyme activity in sheep fed paddy straw based complete feed fortified with probiotics. Biol Rhythm Res. (2022) 53:547–58. doi: 10.1080/09291016.2019.1644019

[30] Ghorbani GR Morgavi DP Beauchemin KA Leedle JAZ. Effects of bacterial direct-fed microbials on ruminal fermentation, blood variables, and the microbial populations of feedlot cattle. J Anim Sci. (2002) 80:1977–85. doi: 10.2527/2002.8071977x, PMID: 12162668

[31] Qiao GH Shan AS Ma N Ma QQ Sun ZW. Effect of supplemental *Bacillus* cultures on rumen fermentation and milk yield in Chinese Holstein cows. J Anim Physiol Anim Nutr. (2010) 94:429–36. doi: 10.1111/j.1439-0396.2009.00926.x, PMID: 19663976

[32] Lascano GJ Heinrichs AJ. Rumen fermentation pattern of dairy heifers fed restricted amounts of low, medium, and high concentrate diets without and with yeast culture. Livest Sci. (2009) 124:48–57. doi: 10.1016/j.livsci.2008.12.007

[33] Wang C Liu Q Zhang YL Pei CX Zhang SL Guo G. Effects of isobutyrate supplementation in pre-and post-weaned dairy calves diet on growth performance, rumen development, blood metabolites and hormone secretion. Animal. (2017) 11:794–801. doi: 10.1017/S1751731116002093, PMID: 27821226

[34] Wei JY Wang J Liu W Zhang KZ Sun P. Effects of different selenium supplements on rumen fermentation and apparent nutrient and selenium digestibility of mid-lactation dairy cows. J Dairy Sci. (2019) 102:3131–5. doi: 10.3168/jds.2018-15455, PMID: 30738681

[35] Cui K Wang Q Wang S Diao Q Zhang N. The facilitating effect of tartary buckwheat flavonoids and *Lactobacillus plantarum* on the growth performance, nutrient digestibility, antioxidant capacity, and fecal microbiota of weaned piglets. Animals. (2019) 9:986. doi: 10.3390/ani9110986, PMID: 31752173 PMC6912274

[36] Long LN Kang BJ Jiang Q Chen JS. Effects of dietary *Lycium barbarum* polysaccharides on growth performance, digestive enzyme activities, antioxidant status, and immunity of broiler chickens. Poult Sci. (2020) 99:744–51. doi: 10.1016/j.psj.2019.10.043, PMID: 32029159 PMC7587896

[37] Dai Z Wang H Liu J Zhang H Li Q Yu X. Comparison of the effects of Yucca saponin, Yucca schidigera, and *Quillaja saponaria* on growth performance, immunity, antioxidant capability, and intestinal flora in broilers. Animals. (2023) 13:1447. doi: 10.3390/ani13091447, PMID: 37174484 PMC10177514

[38] Wang W Wang Y Cui Z Yang Y An X Qi J. Fermented wheat bran polysaccharides intervention alters rumen bacterial community and promotes rumen development and growth performance in lambs. Front. Vet. Sci. (2022) 9:841406. doi: 10.3389/fvets.2022.841406, PMID: 35433917 PMC9007612

[39] Fregulia P Campos MM Dias RJP Liu J Guo W Pereira LGR. Taxonomic and predicted functional signatures reveal linkages between the rumen microbiota and feed efficiency in dairy cattle raised in tropical areas. Front Microbiol. (2022) 13:1025173. doi: 10.3389/fmicb.2022.1025173, PMID: 36523842 PMC9745175

[40] Lesmeister KE Tozer PR Heinrichs AJ. Development and analysis of a rumen tissue sampling procedure. J Dairy Sci. (2004) 87:1336–44. doi: 10.3168/jds.S0022-0302(04)73283-X, PMID: 15290981

[41] Geiger S Patra AK Schrapers KT Braun HS Aschenbach JR. Menthol stimulates calcium absorption in the rumen but not in the jejunum of sheep. J Dairy Sci. (2021) 104:3067–81. doi: 10.3168/jds.2020-19372, PMID: 33358813

[42] Bassareh M Rezaeipour V Abdullahpour R Asadzadeh S. Dietary threonine and lysophospholipid supplement in broiler chickens: effect on productive performance, carcass variables, cecal microbiota activity, and jejunal morphology. Trop Anim Health Prod. (2023) 55:150. doi: 10.1007/s11250-023-03566-8, PMID: 37020151

[43] He C Wu H Lv Y You H Zha L Li Q. Gastrointestinal development and microbiota responses of geese to honeycomb flavonoids supplementation. Front. Vet. Sci. (2021) 8:739237. doi: 10.3389/FVETS.2021.739237, PMID: 34733903 PMC8558617

[44] Tan C Ramírez-Restrepo CA Shah AM Hu R Bell M Wang Z. The community structure and microbial linkage of rumen protozoa and methanogens in response to the addition of tea seed saponins in the diet of beef cattle. J. Anim. Sci. Biotechnol. (2020) 11:1–15. doi: 10.1186/s40104-020-00491-w 32832076 PMC7422560

[45] Belanche A Arturo-Schaan M Leboeuf L Yáñez-Ruiz D Martín-García I. Early life supplementation with a natural blend containing turmeric, thymol, and yeast cell wall components to optimize rumen anatomical and microbiological development and productivity in dairy goats. J Dairy Sci. (2023) 106:4634–49. doi: 10.3168/jds.2022-22621, PMID: 37225586

[46] Samanta AK Jayapal N Senani S Kolte AP Sridhar M. Prebiotic inulin: useful dietary adjuncts to manipulate the livestock gut microflora. Braz J Microbiol. (2013) 44:1–4. doi: 10.1590/S1517-83822013005000023, PMID: 24159277 PMC3804171

[47] Malmuthuge N Griebel PJ Guan LL. The gut microbiome and its potential role in the development and function of newborn calf gastrointestinal tract. Front. Vet. Sci. (2015) 2:36. doi: 10.3389/fvets.2015.00036, PMID: 26664965 PMC4672224

[48] Zhang X Sun Z Cao Y Chen Y Li S Wang G. Effects of dietary inclusion of *Xanthoceras sorbifolia* Bunge leaves on growth performance, gastrointestinal development, digestive function and gut microbial flora of rabbits. Anim Feed Sci Technol. (2022) 292:115438. doi: 10.1016/J.ANIFEEDSCI.2022.115438

[49] Mo J Lu Y Jiang S Yan G Xing T Xu D. Effects of the probiotic, *Lactobacillus delbrueckii* subsp. bulgaricus, as a substitute for antibiotics on the gastrointestinal tract microbiota and metabolomics profile of female growing-finishing pigs. Animals. (2022) 12:1778. doi: 10.3390/ani12141778, PMID: 35883325 PMC9311557

[50] Khalilnia F Mottaghitalab M Mohiti M Seighalani R. Effects of dietary supplementation of probiotic and Spirulina platensis microalgae powder on growth performance immune response, carcass characteristics, gastrointestinal microflora and meat quality in broilers chick. Vet. Med. Sci. (2023) 9:1666–74. doi: 10.1002/VMS3.1154, PMID: 37156247 PMC10357281

[51] Liang J Kou S Chen C Raza SHA Wang S Ma X. Effects of *Clostridium butyricum* on growth performance, metabonomics and intestinal microbial differences of weaned piglets. BMC Microbiol. (2021) 21:85–6. doi: 10.1186/s12866-021-02143-z, PMID: 33752593 PMC7983215

[52] Xue F Wan G Xiao Y Chen C Qu M Xu L. Growth performances, gastrointestinal epithelium and bacteria responses of yellow-feathered chickens to kudzu-leaf flavonoids supplement. AMB Express. (2021) 11:125. doi: 10.1186/S13568-021-01288-4, PMID: 34480270 PMC8417201

[53] Tao W Zhu W Nabi F Li Z Liu J. *Penthorum chinense* Pursh compound flavonoids supplementation alleviates aflatoxin B1-induced liver injury via modulation of intestinal barrier and gut microbiota in broiler. Ecotoxicol Environ Saf. (2023) 255:114805. doi: 10.1016/j.ecoenv.2023.114805, PMID: 36958264

[54] Tamura M Hirayama K Itoh K Suzuki H Shinohara K. Effects of soy protein-isoflavone diet on plasma isoflavone and intestinal microflora in adult mice. Nutr Res. (2002) 22:705–13. doi: 10.1016/S0271-5317(02)00378-0 12350081

[55] Kittelmann S Kirk MR Jonker A McCulloch A Janssen PH. Buccal swabbing as a noninvasive method to determine bacterial, archaeal, and eukaryotic microbial community structures in the rumen. Appl Environ Microbiol. (2015) 81:7470–83. doi: 10.1128/AEM.02385-15, PMID: 26276109 PMC4592876

[56] AlZahal O Li F Walker ND McBride BW. Factors influencing ruminal bacterial community diversity and composition and microbial fibrolytic enzyme abundance in lactating dairy cows with a focus on the role of active dry yeast. J Dairy Sci. (2017) 100:4377–93. doi: 10.3168/jds.2016-11473, PMID: 28390722

[57] Ahmad AA Yang C Zhang J Kalwar Q Liang Z Li C. Effects of dietary energy levels on rumen fermentation. Microbial diversity, and feed efficiency of yaks (*Bos grunniens*). Front Microbiol. (2020) 11:1–2. doi: 10.3389/fmicb.2020.00625 32670204 PMC7326093

[58] Berry D Mader E Lee TK Woebken D Wang Y Zhu D. Tracking heavy water (D2O) incorporation for identifying and sorting active microbial cells. Proc Natl Acad Sci. (2015) 112:E194–203. doi: 10.1073/pnas.1420406112, PMID: 25550518 PMC4299247

[59] Ormerod KL Wood DL Lachner N Gellatly SL Daly JN Parsons JD. Genomic characterization of the uncultured Bacteroidales family S24-7 inhabiting the guts of homeothermic animals. Microbiome. (2016) 4:1–7. doi: 10.1186/s40168-016-0181-2 27388460 PMC4936053

[60] Djemai K Drancourt M Tidjani Alou M. Bacteria and methanogens in the human microbiome: a review of syntrophic interactions. Microb Ecol. (2022) 83:536–54. doi: 10.1007/S00248-021-01796-7, PMID: 34169332

[61] Ma T Chen DD Tu Y Zhang NF Si BW Diao QY. Dietary supplementation with mulberry leaf flavonoids inhibits methanogenesis in sheep. Anim Sci J. (2017) 88:72–8. doi: 10.1111/asj.12556, PMID: 27112278

[62] Kozłowska M Cieślak A Jóźwik A El-Sherbiny M Stochmal A Oleszek W. The effect of total and individual alfalfa saponins on rumen methane production. J Sci Food Agric. (2020) 100:1922–30. doi: 10.1002/jsfa.10204, PMID: 31846083

[63] Qi M Cao Z Shang P Zhang H Hussain R Mehmood K. Comparative analysis of fecal microbiota composition diversity in Tibetan piglets suffering from diarrheagenic *Escherichia coli* (DEC). Microb Pathog. (2021) 158:105106. doi: 10.1016/j.micpath.2021.105106, PMID: 34311015

[64] Sadeghpour Heravi F Hu H. Bifidobacterium: host–microbiome interaction and mechanism of action in preventing common gut-microbiota-associated complications in preterm infants: a narrative review. Nutrients. (2023) 15:709. doi: 10.3390/NU15030709, PMID: 36771414 PMC9919561

[65] Shang Q Wang Y Pan L Niu Q Li C Jiang H. Dietary polysaccharide from *Enteromorpha clathrata* modulates gut microbiota and promotes the growth of *Akkermansia muciniphila*, Bifidobacterium spp. and Lactobacillus spp. Mar Drugs. (2018) 16:167. doi: 10.3390/md16050167, PMID: 29772753 PMC5983298

[66] Chen G Li Z Liu S Tang T Chen Q Yan Z. Fermented Chinese herbal medicine promoted growth performance, intestinal health, and regulated bacterial microbiota of weaned piglets. Animals. (2023) 13:476. doi: 10.3390/ani13030476, PMID: 36766365 PMC9913397

[67] Tian Q Wu H Wang C Lu W Cai B. Effects of fermented Chinese herbal medicine oral liquid on growth performance, blood routine, serum biochemical, serum immune indexes, and fecal microbiota of puppies. Feed Res. (2025) 48:145–9. doi: 10.13557/j.cnki.issn1002-2813.2025.09.025

